# Spleen rates and infant parasite rates as surveillance tool for malaria control in remote hard to reach areas of central India

**DOI:** 10.1186/1475-2875-10-381

**Published:** 2011-12-21

**Authors:** Manmohan Shukla, Neeru Singh, Mrigendra P Singh

**Affiliations:** 1 National Institute of Malaria Research, Field Station, Jabalpur 482003, Madhya Pradesh, India; 2 Regional Medical Research Centre for Tribals (ICMR), Nagpur Road, Garha, Jabalpur 482003, Madhya Pradesh, India

## Abstract

**Background:**

Malaria due to both *Plasmodium falciparum *and *Plasmodium vivax *is a major public health problem in India. The quantification of malaria transmission for the classification of malaria risk has long been a concern for epidemiologists. Results are presented from 30 cross-sectional surveys which measured spleen rates (SR) and infant parasite rates (IPR) in the forested districts of Madhya Pradesh during malaria outbreaks to assess whether both IPR and SR can still be used as indicators of malaria endemicity as spleen examination has lost much of its value as an epidemiological indicator in areas where anti-malarials drugs are widely used.

**Methods:**

Rapid fever surveys were carried out from door to door and all suspected malaria cases in the entire population of a village were screened for malaria parasites on the basis of clinical symptoms such as fever, chill, rigor, headache and body ache etc. Children between 2 and 9 years were examined for enlarged spleen according to Hacketts method. Finger prick blood smears were collected from all children with enlarged spleen with or without fever after obtaining written informed consent following institutional ethical guidelines. Infants less than 1 year were also screened for malaria with or without fever.

**Results:**

Since malaria is local and focal, in some areas the outbreak waned quickly in few months and in some areas continued for 3 to 4 years. The analysis of trend revealed that when IPR decline over the years as a result of malaria intervention measures, SR also decline. In case splenomegaly continues without diminution in size, it is probably due to recrudescence or relapse, although it is not possible to separate malaria parasite species on the basis of SR.

**Conclusion:**

Both the tools are of immense value in evaluating and assessing the malaria situation especially in remote areas where sophisticated molecular and serological techniques are difficult to establish. Therefore, in forested areas malaria surveillance system will require adoption of multiple approaches that have proven effective now or in the past.

## Background

Malaria in India is classically considered to be unstable and prone to epidemics [1]. Central India is a highly malarious state in India with sizeable population at risk, which hides extreme variations in terms of transmission settings. Little is known about the epidemic periodicity of malaria in India and the average duration of epidemic events [2]. The epidemic period is highly variable depending on the intervention measures and host immunity. Even if malaria is significantly controlled in some states in India, frequent migration would give ample opportunity for re-emergence. Consequently, malaria flares up from one place to another whenever favourable ecological conditions prevail and further defuses. This unstable malaria transmission makes control extremely challenging. This places special emphasis on the disease surveillance system as quantification of malaria transmission for the classification of malaria risk has long been a concern for epidemiologists [3]. Use of splenomegaly for epidemiological assessment of malaria is an age-old practice [4]. Even before the discovery of the malaria parasite by Laveran in 1880, Dempster pointed out the significance of splenomegaly in malaria in 1848 [5]. However, spleen examination has lost much of its value as an epidemiological indicator in areas where anti-malarial drugs are very widely used [6]. Similarly infant parasite rate is a good indicator of malaria prevalence in an area [7,8]. However, sophisticated molecular and serological techniques are nowadays the latest trend in spite of difficulty in their routine application in running a control programme.

**Figure 1:**
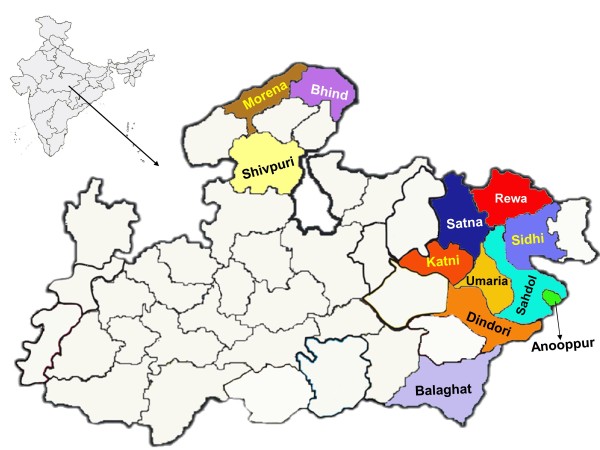
**Map of India showing Madhya Pradesh State and districts where malariometric surveys carried out**.

During 2005-2011, several malaria outbreaks were investigated by Regional Medical Research Centre for Tribals (RMRCT) in inaccessible forested villages of many districts where data on enlarged spleen from young children with or without symptoms and blood smears from fever cases and cases with history of fever were collected from all age groups from non-endemic to meso-endemic districts (Figure 1). In this study, the available data on enlarged spleen and blood smears from infants were analyzed to assess whether spleen rate (SR) and infant parasite rate (IPR) can still be used as epidemiological tools to evaluate prevailing malaria situation and the impact of intervention measures.

## Methods

### Study area and population

Madhya Pradesh (central India) is in the central part of India with an area of 308,000 km^2^, of which forest covers 76,429 km^2 ^(about 25% of the total land area). Low and unstable malaria transmission and intermittent epidemics are common throughout the state. The malariogenic potential is very high because of the presence of two efficient vectors, insecticide resistance, presence of both *Plasmodium vivax *and *Plasmodium falciparum *and intensifying anti-malarial drug resistance.

A total of 60 cross-sectional surveys were carried out in 20 districts of the State during malaria outbreaks on the request of government of Madhya Pradesh of which data of only twelve districts (30 surveys) are discussed here. These 30 surveys are randomly selected out of 12 districts using SPSS 17 for Windows. Most of the affected Primary Health Centres (PHCs) are in forest and inhabitants are mostly ethnic tribe. These PHCs are under two rounds of DDT indoor residual spray. The villages are interspersed with streams and their tributaries. The streams are prone to frequent floods during rains, which disrupt communication for several months. Most adults work in field or forest nurseries or on forest road construction and maintenance. The houses are dark, damp and made of wood without ventilation and electricity.

Rapid fever surveys were carried out from door to door in the entire population of a village and all suspected malaria cases on the basis of clinical symptoms, such as fever, chill, rigour, headache and body ache, were screened for malaria parasites. Children between 2 and 9 years with or without fever were examined for enlarged spleen according to Hacketts method [9]. Finger prick blood smears were collected from all children with enlarged spleen after obtaining written informed consent following institutional ethical guidelines. Infants less than one year were also screened for malaria with or without fever. Blood films were stained with Jaswant Singh and Bhattacharji stain [10]. For quality control, all slides were re-examined by a single microscopist who was unaware of the previous results. All parasite positive cases were administered anti-malarials following current recommendations of National Vector Borne Disease Control Programme (NVBDCP).

### Data analysis

Data was entered in Epi Info 3.5.1 CDC, Atlanta and SPSS 17 for Windows was used for data analysis. The spleen rate (SR) was computed by dividing the number of children with enlarged spleen by the total number of children examined. The average enlarged spleen (AES) index for each survey was obtained by the sum of the number of children in each spleen size class multiplied by the class number (0-5) divided by the total number of palpable spleen. The slide positivity rate (SPR) refers to the proportion of malaria positive blood smears among all smears. Fever was defined as an auxiliary temperature ≥ 37.5°C.

## Results

**Figure 2:**
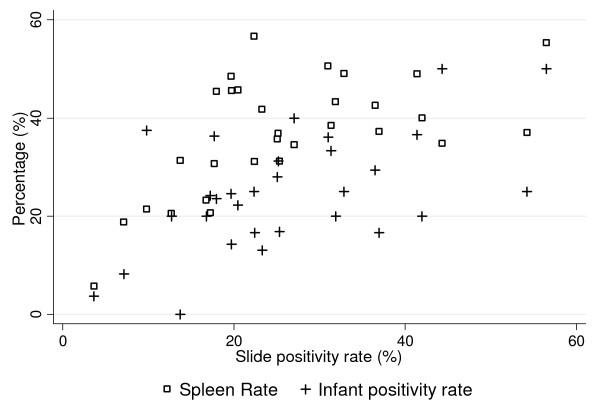
**Scatter plot showing correlation of Slide positivity rate in all age group (SPR) with Spleen rate (SR) and Infant parasite rate (IPR)**.

Additional file 1: Table S1 shows the district and year of the survey, the number of children examined for spleen, the number of enlarged spleen and AES, number of malaria positives among splenomegaly cases, the number of blood smear examined from infants and symptomatic older age groups covering entire population of the village (pooled) and the number of blood smear positive to any species of malaria parasite. These results reflect that both SR and IPR were very high during outbreaks as seen in districts Balaghat, Dindori, Morena, Satna, Sheopur, Shivpuri, Sidhi and Anuppur followed by a gradual decline after the initiation of intervention measures. Spleen enlargement is due to both *P. vivax *and *P. falciparum*, although it is not possible to separate malaria parasite species on the basis of SR. Interestingly, in Sidhi and Balaghat, high SR and IPR were found consistently for 5 and 4 years respectively, while in Dindori high SR and IPR were found for 3 years inspite of intervention measures. On the other hand, the corresponding values in most districts were high for 2 years and in some districts for 1 year indicating local variations. Further, when transmission is decreased as compared to previous years SR and IPR also showed corresponding changes such as Satna. Furthermore, SR and IPR were directly proportional to the overall SPR in general febrile population covering all age groups except in few districts such as Anuppur, Sheopur, which could be due to local differences in the population and overall immune response to the parasites. Figure 2 shows the positive association between SPR in all age group in general population and SR (r = 0.59 *P *< 0.001) and SPR and IPR (r = 0.55, *P *< 0.0025).

## Discussion

Malaria is a major public health problem in India even though it is both preventable and treatable disease [11]. Malaria presents a diagnostic challenge as *P. falciparum *can present with a wide spectrum of signs, symptom and history from a fatal disease to an apparently asymptomatic infection, from a rapidly progressing fulminate illness to a chronic insult [12]. Further, malaria produces anaemia by several mechanisms. Both acute *P. falciparum *and chronic or recurrent low level parasitaemia can produce hemolysis [13]. In forest villages where resources for malaria diagnosis are limited, malaria diagnosis is mostly made on the basis of clinical symptoms although this is alarmingly inaccurate [14]. The detection of *P. falciparum*/*P. vivax *can be made on the spot by recently introduced rapid diagnostic tests in symptomatic patients with fairly high sensitivity and specificity [15]. However, it is not always available in remote areas [16]. Spleen enlargement, fever and anaemia are the three main signs characteristic of malaria infection. Children with malaria had a lower haematocrit than aparasitaemic children. Therefore, measure of haematocrit index for anaemia could also be useful in field along with surveillance of fever cases [17,18]. One of the most striking findings of the study was high SR and IPR in all the outbreak-affected areas indicating intense malaria transmission. Both the indices are of immense value in evaluating and assessing the malaria situation in an area. While the former is a crude method and reflects the endemicity of the area, the latter is an indicator of recent transmission of malaria. It is worthwhile to mention that surveillance data on epidemic is often not collected because epidemics may be over before health services have had time to intervene or because in severe situations, reporting procedures may breakdown. Further, once achieved, malaria control cannot be taken for granted and must be actively maintained. The rapid re-introduction of cases from neighbouring areas is very common. Both SR and IPR can quickly track the effects of intervention packages for malaria control as recorded earlier [7,8,19]. They are of great diagnostic aid as these could serve to identify scattered endemic villages in hypo-endemic areas which maintain the parasite reservoir during the inter-epidemic periods and from which explosive epidemics may spread [6]. These tools can also be applied in malaria control programmes, in advance elimination or even in the maintenance phase [7,8,18]. In fact, the spleen examination was even applied in advanced malaria eradication programmes and in maintenance phase in different geographical location very efficiently [20]. The enlarged spleen without much diminution in size, in most districts, is due to frequent recrudescence or relapses such as in Balaghat, Sidhi and Dindori. Further, Thomas et al [21] found a positive correlation between seroepidemiological study and spleen results in aborigine children in Malaysia where both *P. vivax *and *P. falciparum *were prevalent.

The current global strategy for malaria elimination requires surveillance tools for the evaluation of accomplishment at the different geographical scales of the elimination goals [22]. Indicators derived from surveillance tools need to be precise, accurate and representative of changes in transmission patterns at the population level [23]. The utilization of the spleen examination and infant parasite rate as a tool of malaria evaluation is considered by the authors as one of the most valuable, practical, simple and economical method which yield immediate and practical results in several malarial outbreaks [24,25]. However, De et al [4] detected splenomegaly in only 13% malaria patients which does not signify a positive correlation between splenomegaly and malaria, perhaps due to widespread use of anti-malarials.

The new methods of detection of low numbers of asexual and sexual stages of parasites by molecular techniques [26,27] and new approaches to serology [28] could have an important role in surveillance. However, they are difficult to establish in rural/semi rural laboratories by inexperienced technicians. They are of tremendous value in research projects. [20].

## Conclusion

Finally, in remote and inaccessible areas, if one uses SR and IPR in the field, it would be easy to have an idea of the malaria history of the area, the present epidemiological situation and the impact of intervention measures. Thus, it is now amply clear that the disease surveillance system in forested areas will require adoption of multiple approaches that have proven effective now or in the past.

## Abbreviations

SR: Spleen rates; IPR: Infant parasite rates; SPR: Slide positivity rates; PHCs: Primary health centres; NVBDCP: National vector borne disease control programme; AES: Average enlarged spleen

## Competing interests

The authors declare that they have no competing interests.

## Authors' contributions

MMS performed spleen examination of children and infant parasite survey in field. NS developed the concept, study design and participated in manuscript preparation. MPS performed data analysis and result interpretation. All authors read and approved the final manuscript.

## Supplementary Material

Additional file 1 **Table S1**. Malariometric surveys in various districts of Madhya Pradesh showing malaria endemicity Click here for file
