# Supplementary material for: Spleen rates and infant parasite rates as surveillance tool for malaria control in remote hard to reach areas of central India
Source: Malar J. 2011 Dec 21;10:381. doi: 10.1186/1475-2875-10-381 (PMC3258211; doi:10.1186/1475-2875-10-381)
Supplement: Additional file 1 — Table S1. Malariometric surveys in various districts of Madhya Pradesh showing malaria endemicity [file 1475-2875-10-381-S1.DOC]

**Table S1: Malariometric surveys in various districts of Madhya Pradesh showing malaria endemicity**

| **Year** | **District** | **Population covered** | **Spleen examination** | | | | **Malaria in spleenomegaly cases** | | | **Malaria in rapid fever survey** | | | | | **Malaria in infants** | | | | |
| --- | --- | --- | --- | --- | --- | --- | --- | --- | --- | --- | --- | --- | --- | --- | --- | --- | --- | --- | --- |
|  |  |  | **Examined** | **+ve** | **SR** | **AES** | **+ve** | **Pv** | **Pf** | **Examined** | **+ve** | **Pv** | **Pf** | **SPR** | **Examined** | **+ve** | **Pv** | **Pf** | **IPR** |
| 2005 | KATNI | 7540 | 220 | 88 | 40 | 1.75 | 35 | 15 | 20 | 412 | 173 | 51 | 122 | 41.99 | 5 | 1 | 0 | 1 | 20 |
| 2006 | KATNI |  | 78 | 30 | 38.46 | 2 | 18 | 5 | 13 | 536 | 168 | 14 | 154 | 31.34 | 3 | 1 | 0 | 1 | 33.33 |
| 2007 | KATNI |  | 35 | 16 | 45.71 | 2.06 | 2 | 1 | 1 | 313 | 64 | 21 | 43 | 20.45 | 9 | 2 | 0 | 2 | 22.22 |
| 2008 | MORENA | 8315 | 115 | 49 | 42.61 | 2 | 26 | 0 | 26 | 1474 | 538 | 34 | 504 | 36.5 | 17 | 5 | 2 | 3 | 29.41 |
| 2009 | MORENA |  | 45 | 14 | 31.11 | 1.93 | 3 | 1 | 2 | 357 | 80 | 28 | 52 | 22.41 | 6 | 1 | 0 | 1 | 16.67 |
| 2005 | SATNA | 20580 | 390 | 191 | 48.97 | 2.02 | 92 | 20 | 72 | 2411 | 998 | 94 | 904 | 41.39 | 71 | 26 | 6 | 20 | 36.62 |
| 2006 | SATNA |  | 1315 | 410 | 31.18 | 1.9 | 112 | 82 | 30 | 1919 | 486 | 237 | 249 | 25.33 | 77 | 13 | 6 | 7 | 16.88 |
| 2007 | SATNA |  | 610 | 35 | 5.74 | 1.2 | 5 | 3 | 2 | 55 | 2 | 0 | 2 | 3.64 | 27 | 1 | 0 | 1 | 3.7 |
| 2008 | SHIVPURI | 18900 | 733 | 371 | 50.61 | 1.95 | 148 | 33 | 115 | 1449 | 449 | 94 | 355 | 30.99 | 36 | 13 | 1 | 12 | 36.11 |
| 2009 | SHIVPURI |  | 75 | 23 | 30.67 | 2.04 | 3 | 1 | 2 | 1671 | 296 | 115 | 181 | 17.71 | 11 | 4 | 2 | 2 | 36.36 |
| 2006 | SIDHI | 28500 | 180 | 78 | 43.33 | 1.9 | 44 | 12 | 32 | 797 | 254 | 38 | 216 | 31.87 | 25 | 5 | 2 | 3 | 20 |
| 2007 | SIDHI |  | 550 | 270 | 49.09 | 2.05 | 95 | 2 | 93 | 2821 | 927 | 101 | 826 | 32.86 | 24 | 6 | 0 | 6 | 25 |
| 2008 | SIDHI |  | 510 | 289 | 56.67 | 1.99 | 107 | 5 | 102 | 2180 | 487 | 49 | 438 | 22.34 | 12 | 3 | 0 | 3 | 25 |
| 2009 | SIDHI |  | 180 | 82 | 45.56 | 2.01 | 38 | 2 | 36 | 381 | 75 | 2 | 73 | 19.69 | 14 | 2 | 0 | 2 | 14.29 |
| 2010 | SIDHI |  | 30 | 11 | 37.0 | 2.0 | 0 | 0 | 0 | 201 | 109 | 3 | 106 | 54.23 | 8 | 2 | 0 | 2 | 25.00 |
| 2008 | BALAGHAT | 18600 | 610 | 142 | 23.28 | 2 | 37 | 4 | 33 | 758 | 127 | 21 | 106 | 16.75 | 20 | 4 | 1 | 3 | 20 |
| 2009 | BALAGHAT |  | 175 | 61 | 34.86 | 2 | 8 | 0 | 8 | 404 | 179 | 9 | 170 | 44.31 | 8 | 4 | 1 | 3 | 50 |
| 2010 | BALAGHAT |  | 1215 | 589 | 48.48 | 2.01 | 195 | 16 | 179 | 3023 | 594 | 101 | 493 | 19.65 | 183 | 45 | 15 | 30 | 24.59 |
| 2011 | BALAGHAT |  | 1436 | 295 | 20.54 | 1.99 | 73 | 19 | 54 | 977 | 124 | 30 | 94 | 12.69 | 40 | 8 | 2 | 6 | 20 |
| 2008 | BHIND | 11620 | 210 | 75 | 35.71 | 1.85 | 1 | 1 | 0 | 3656 | 916 | 282 | 634 | 25.05 | 25 | 7 | 3 | 4 | 28 |
| 2009 | BHIND |  | 56 | 12 | 21.43 | 1.83 | 8 | 6 | 2 | 950 | 93 | 51 | 42 | 9.79 | 8 | 3 | 3 | 0 | 37.5 |
| 2009 | DINDORI | 22500 | 275 | 95 | 34.55 | 1.95 | 45 | 8 | 37 | 2417 | 653 | 82 | 571 | 27.02 | 85 | 34 | 8 | 26 | 40 |
| 2010 | DINDORI |  | 300 | 62 | 20.67 | 1.82 | 24 | 6 | 18 | 2398 | 413 | 94 | 319 | 17.22 | 161 | 39 | 17 | 22 | 24.22 |
| 2011 | DINDORI |  | 975 | 183 | 18.77 | 1.99 | 24 | 5 | 19 | 1726 | 123 | 29 | 94 | 7.13 | 85 | 7 | 2 | 5 | 8.24 |
| 2010 | ANUPPUR | 15800 | 160 | 59 | 36.88 | 2 | 24 | 4 | 20 | 298 | 75 | 8 | 67 | 25.17 | 16 | 5 | 0 | 5 | 31.25 |
| 2011 | ANUPPUR |  | 434 | 197 | 45.39 | 1.96 | 53 | 8 | 45 | 206 | 37 | 8 | 29 | 17.96 | 17 | 4 | 1 | 3 | 23.53 |
| 2006 | SHAHDOL | 8530 | 542 | 227 | 41.80 | 2.0 | 77 | 23 | 54 | 760 | 177 | 36 | 141 | 23.29 | 23 | 3 | 1 | 2 | 13.04 |
| 2005 | UMERIYA | 6330 | 168 | 93 | 55.35 | 2.0 | 54 | 13 | 41 | 138 | 78 | 19 | 59 | 56.52 | 2 | 1 | 1 | 0 | 50.00 |
| 2006 | UMERIYA |  | 204 | 64 | 31.37 | 2.0 | 15 | 1 | 14 | 408 | 56 | 14 | 42 | 13.73 | 6 | 0 | 0 | 0 | 0.00 |
| 2011 | REWA | 6530 | 145 | 54 | 37.24 | 1.9 | 22 | 1 | 21 | 230 | 85 | 29 | 56 | 36.96 | 6 | 1 | 1 | 0 | 16.67 |

SR: Spleen Rate, AES: Average Enlarged Spleen, +ve: Malaria positive cases, Pv: *P. vivax*, Pf: *P. falciparum*, SPR: Slide Positivity Rate, IPR: Infant Parasite Rate
